# Morpheme-Based Reading and Writing in Spanish Children with Dyslexia

**DOI:** 10.3389/fpsyg.2017.01952

**Published:** 2017-11-07

**Authors:** Paz Suárez-Coalla, Cristina Martínez-García, Fernando Cuetos

**Affiliations:** Department of Psychology, University of Oviedo, Oviedo, Spain

**Keywords:** morphology, reading, spelling, dyslexia, Spanish

## Abstract

It has been well documented that morphemic structure (roots and affixes) have an impact in reading, but effects seem to depend on the reading experience of readers and lexical characteristics of the stimuli. Specifically, it has been reported that morphemes constitute reading units for developing readers and children with dyslexia when they encounter a new word. In addition, recent studies have stated that the effect of morphology is also present in spelling, as morphological information facilitates spelling accuracy and influences handwriting times. The goal of this study was to investigate the role of morphology in reading and spelling fluency in Spanish children with dyslexia. For that purpose, a group of 24 children with dyslexia was compared with an age-matched group of 24 children without reading disabilities in performing a word naming task and a spelling-to-dictation task of isolated words. Morphological condition (high frequency base, low frequency base, simple) and lexicality (words vs. pseudowords) were manipulated. We considered, for the naming task, reading latencies, reading durations, reading critical segment (three first phonemes) durations and naming accuracy; and, for the spelling task, written latencies, writing durations for the whole word, writing critical segment (three first letters) durations and spelling accuracy. Results showed that Spanish children (with and without dyslexia) benefit from a high frequency base to initiate reading and writing responses, showing that they are familiar with the letter chunks that constitute a morpheme. In addition, base frequency impacts reading critical segment duration only for children with dyslexia, but for both groups in writing. In summary, children with dyslexia benefit from a high frequency base to read and spell unfamiliar stimuli.

## Introduction

Developmental dyslexia is characterized by specific and pronounced difficulties in learning to read and spell which are unexpected considering the fact that the child’s cognitive abilities and educational opportunities are within the normal range in relation to their peers (Lyon et al., 2003). Children with dyslexia are prone to make reading and spelling mistakes and show reading speed deficits (Ziegler et al., 2003); however, these characteristics appear to be determined by the orthographic depth. In transparent orthographies (e.g., Spanish or Italian), in contrast to the opaque ones, the more prominent problem in dyslexia is reading speed rather than reading accuracy (Wimmer, 1993; Suárez-Coalla and Cuetos, 2012). Considerable accuracy in transparent orthographies is explained by consequence of the spelling-sound consistency (Seymour et al., 2003), while slow reading speed is considered a consequence of problems in automating the alphabetic code and in achieving the orthographic representations of words (Manis, 1985; Bergmann and Wimmer, 2008; Suárez-Coalla et al., 2014). As a result, Spanish children with dyslexia have special difficulties with long low frequency words and long pseudowords (Davies et al., 2007, 2013; Suárez-Coalla and Cuetos, 2012), indicating that these children rely on small grain-size units of the words (Davies et al., 2013; Suárez-Coalla et al., 2014). In addition, spelling difficulties have been reported in Spanish children with dyslexia, as they present more mistakes than typically developing children when spelling (Suárez-Coalla et al., 2016). Moreover, like in other consistent orthographies (e.g., in Italian: Angelelli et al., 2004, 2010), children with dyslexia show a specific pattern of errors, as they make a high number of phonologically plausible errors in words with unpredictable spelling, indicating the reliance on a sublexical strategy (Suárez-Coalla et al., 2016).

On the other hand, morphemes constitute an intermediate unit, between the letter and the word, with impact on reading and spelling (Burani et al., 2002; Kandel et al., 2008; Lázaro et al., 2013; Quémart and Casalis, 2016). It is well established that morphology in opaque orthographic systems is very useful for assigning the correct pronunciation and spelling of words (Carlisle and Stone, 2005), but some effects of morphology have also been reported in transparent orthographies, indicating that morphemes are useful intermediate units in every kind of orthographic system (Burani et al., 2002). Nevertheless, morphological effects appear to be determined by task, stimulus characteristics (lexical frequency, length, morpheme type) and literacy experience. Specifically, people with dyslexia are able to recognize and rely on the morphemic structure. Although there are not many studies about this topic in Spanish (and other shallow orthographies), some results support the hypothesis that morphemic units (larger than the grapheme and smaller than the word) could assist reading and spelling processes in this population.

Burani et al. (2002) showed that Italian children (8–10 years old), with and without dyslexia, benefit from morphological structure to read pseudowords (made up of roots: DONN- ‘woman’ and derivational suffixes: -ISTA, ‘ist’), as they were named faster and more accurately than matched pseudowords with no morphological structure (e.g., DENNOSTO); the same benefit happens with adults. When the stimuli were words (e.g., derived: CASSIERE, ‘cashier’ vs. simple: CAMMELLO, ‘camel’), only children with dyslexia and young readers benefited from morphology, so morphological structure did not impact word naming of skilled readers (Burani et al., 2008). On the other hand, it has been confirmed that the impact of morphological structure in word naming is modulated by word frequency and reading skills, as young skilled readers only took advantage of morphology when reading low frequency words, while poor readers benefited in both high and low frequency words, and adults did not benefit in any case (Marcolini et al., 2011). These results suggest that morpheme-based reading in transparent orthographies appears in the absence of well specified orthographic representation (for poor readers, or in low frequency words and pseudowords), which implies that after a complex word has been frequently processed, the orthographic representation for the whole word will have a more important role than its component morphemes.

Additionally, the frequency of morphemes was also found to modulate the use of morphology in Italian children with dyslexia-dysorthography and typically developing children. In a study by Angelelli et al. (2017), third grade children had to read three sets of words (1: derived with high frequency morphemes, 2: derived with low frequency morphemes, 3: non-derived) and two sets of pseudowords (1: combining root + suffix, 2: non-derived). They found that morphology facilitated pseudoword reading accuracy in both groups; in addition, both groups took advantage of high frequency morphemes when reading low frequency words, but, surprisingly, children with dyslexia showed worse results in words with low frequency morphemes than in simple ones.

Regarding the Spanish language, a transparent orthography, only two studies have to our knowledge addressed the role of morphology in reading of children with dyslexia (Lázaro et al., 2013; Suárez-Coalla and Cuetos, 2013). Lázaro et al. (2013) explored the effect of base frequency (i.e., frequency of the target word stem) in a lexical decision task on the supposition that children are able to process morphemic structure, so complex words with a high frequency base could elicit faster responses than low frequency base words. They found that only older skilled children in the study (8 years, age-matched to children with reading problems) showed the facilitation effect of base frequency. In contrast, children with dyslexia did not take advantage of morphology in reaction times. However, Suárez-Coalla and Cuetos (2013) found that children with dyslexia benefited from morphology, since they named complex stimuli (e.g., word: BELL-EZA, ‘beaut-y’; pseudoword: PLAT-EZA) better than simple ones (e.g., word: PEREZA, ‘laziness’; pseudoword: ASTOZA). These latter results agree with the hypothesis that morphological processing constitutes a compensatory strategy for people with reading difficulties by recognizing units larger than the grapheme. As mentioned before, children with dyslexia show difficulties in developing orthographic representation at the whole word level. Therefore the development of morphological representations (units of multiple grain size) would allow smooth and precise reading, especially with unknown words. In this sense, the morphological processing would be a complementary strategy to grapheme–phoneme conversion and lexical reading. This kind of strategy fits with the recent conceptualization of dual-route models (Grainger and Ziegler, 2011), where lexical route is supposed to include indirect access via letter co-occurrences (e.g., morphemes). In the latter work, words were matched in superficial frequency, but base frequency was not considered, a variable that seems to impact on the visual recognition of words, as suggested by Lázaro et al. (2013). In consequence, it will be possible to find effects of root frequency in word naming.

As in reading, a considerable number of researches in opaque orthographies (French and English) reported benefits of morphology in spelling with accuracy in typically developing children (Treiman et al., 1994; Sénéchal, 2000; Deacon and Bryant, 2006; Kemp, 2006; Pacton and Deacon, 2008; Bourassa and Treiman, 2009; Casalis et al., 2011; Pacton et al., 2013; Quémart and Casalis, 2016) and children with dyslexia (Bourassa et al., 2006; Quémart and Casalis, 2016). Morphological constancy (spelling maintenance of root in family words) helps in spelling words properly despite the phonological changes that may occur (e.g., in English: HEALTH: /hɛlθ/ vs. HEAL: /hiːl/), even from an early age. In more transparent orthographies, morphological effects have also been reported for children with no difficulties (Finnish: Lehtonen and Bryant, 2005; Spanish: Défior et al., 2008; Italian: Angelelli et al., 2014) and with dyslexia (Diamanti et al., 2014; Angelelli et al., 2017). Besides, as for reading, effects look to be modulated by the frequency of the morphological constituents. Benefits were found in low frequency words with high frequency morphemes, while words with low frequency morphemes achieved a similar performance to non-derived words (Angelelli et al., 2017). According to those results, children with dyslexia use some morphological information when spelling, evident in accuracy, by retrieving pre-assembled units.

Recently, new techniques (mainly digitizer tablets) are being used to investigate writing processes, allowing the collection of chronometric measures and providing interesting results about the role of morphology in spelling. In this vein, some data support that morpheme-sized units play a role in handwriting production (Orliaguet and Boë, 1993; Kandel et al., 2008; Kandel et al., 2012). Kandel et al. (2012) observed that movement duration of the root was longer for suffixed words than for pseudo-suffixed words, suggesting that suffix programming is not fully completed when the motor response starts, implying extra time during root production. According to this, a new psycholinguistic model of handwriting was proposed by Kandel et al. (2011), where the orthographic representation of a word could imply a multidimensional structure. This model considers a spelling module (after a linguistic module and before a motor module), where different kinds of abstracts processing levels (words, morphemes, syllables, concurrent letters and letters) exist and could be active in parallel. In this sense, handwriting production would involve the activation of intermediate units (morphemes) between syllables and words (Kandel et al., 2012). The mentioned methodology offers one opportunity to improve knowledge about the role of morphology in children with dyslexia.

Taking into consideration the reviewed literature about morphological effects, we have arguments to hypothesize that morphological structure could play a significant role in reading and writing in transparent orthographic systems, with recognizable effects in accuracy and speed. It could be supposed that morpheme-based reading and spelling will appear for Spanish children with dyslexia, as it has been suggested that the role of morphology is more evident in the absence of orthographic representation. Specifically, we assume greater accuracy and fluency in reading and spelling morphologically complex stimuli than simple ones. However, the facilitation effect could, at the same time, be determined by variables such as lexicality, lexical frequency and base frequency (root frequency), apart from the type of morpheme or the length of the word.

In this framework, the present study addressed the role of morphology in both reading and spelling in Spanish children with dyslexia. We aimed to know if children with dyslexia take advantage of morphology, and whether or not facilitation depends on the root frequency. The facilitation effect of morphology in children with dyslexia would indicate that they are able to use intermediate units when they do not have robust orthographic representation of a word, constituting a compensatory strategy. Children with and without dyslexia completed a word naming task and a spelling-to-dictation task of isolated words and pseudowords, where morphological condition (high frequency base, low frequency base, and simple) was manipulated. The inclusion of pseudowords, made from a real root, constitutes a guarantee that participants do not have orthographic representations of the whole stimuli, and use of morphological processing could be evident. The analysis considered, for the naming task, naming accuracy, reading latencies (RL), reading durations (RD) and reading critical segment durations (RCSD; the first three phonemes, which correspond to the root in morphologically complex words); and for the spelling-to-dictation task, spelling accuracy, written latencies (WL), writing durations (WD) for the whole word and writing critical segment durations (WCSD; the first three letters, corresponding to the root in complex words). We expected that morphological knowledge, particularly base frequency, would have an impact on reading and spelling times, especially in children with dyslexia when they face pseudowords.

## Materials and Methods

### Participants

Twenty-four children with developmental dyslexia (ages 7–12: *M* = 9.9, *SD* = 1.5) and 24 controls (ages 7–12: *M* = 9.9, *SD* = 1.45) participated in this study. Children were recruited from several primary schools in Asturias (Spain) and were matched by age and gender (12 females and 12 males per group). All the participants were native Spanish speakers and had no known motor or perceptual disorders. Participants with dyslexia (DYS) had an IQ of 85 or higher (*M* = 103, *SD* = 12) according to the Wechsler Intelligence Scale for Children (WISC, Wechsler, 2001) and showed persistent reading problems. (It should be noted that, in Spain, children start reading instruction at 4 years old and at around 6 years every child presents a high level of reading accuracy.) Children with dyslexia were given an extended number of tests (e.g., reading process, writing process, phonological awareness, working memory, etc.) and WISC was used to confirm that reading problems were not due to general cognitive problems. Their profile satisfied the criteria of the International Association of Dyslexia (cited in Shaywitz, 2003): (a) reading skills are highly deviant in relation to the control matched by age (*SD* = 1.5–2); (b) persistent reading problem, despite instruction and training; and (c) reading problem more severe than expected based on intellectual capacity and socio-economic status. Control children did not show any type of learning disability according to the assessment of the school psychologist. The reading level of participants was taken from the test battery PROLEC-R (Cuetos et al., 2007) in order to confirm the reading problems of children with dyslexia. PROLEC-R gives scores for word and pseudoword reading. The word reading subtest includes 40 Spanish words (high frequency [HF], and low frequency [LF], short and long words). The pseudoword reading section consists of 40 stimuli (short and long). Children included in the group with dyslexia showed accuracy and/or reading speed scores two standard deviations (SD) below the age mean according to norms provided by PROLEC-R. Children in the control group (CON) had a reading level in line with their age in both measures. Means, standard deviations and *p-*values for demographic characteristics and scores obtained in reading assessment tests are provided in **Table 1**.

**Table 1:** Means and standard deviations (in parenthesis) for reading scores of children with dyslexia and controls.

	Control group	Dyslexia group	*p-*values
	mean (*SD*)	mean (*SD*)	
Age (years)	9.9 (1.55)	9.9 (1.58)	*p* = 0.78
**Reading words**			
Accuracy	39.60 (0.70)	37.21 (9.71)	*p* = 0.24
Speed (s)	33.60 (14.38)	74.83 (34.78)	*p* < 0.001
**Pseudowords**			
Accuracy	38.9 (1.22)	30.90 (5.40)	*p* < 0.001
Speed (s)	51.46 (34.78)	91.13 (24.75)	*p* < 0.001

### Materials

Thirty-six stimuli were selected, comprising 18 words and 18 pseudowords. All stimuli had six letters and three syllables, where morphological condition (morphologically complex with HF base, morphologically complex with LF base and simple words) and lexicality were manipulated in order to create six experimental sets with six stimuli in each set. The words (three sets) were matched by superficial lexical frequency according to the LEXESP database (Sebastián-Gallés et al., 2000). Words in the first set (e.g., PELUDO, ‘hairy’) consisted of an HF root (PEL-), and a derivational suffix, (-UDO); words in the second set (e.g., TEJADO, ‘roof’), consisted an LF root (TEJ-), and a derivational suffix, (-ADO); words in the third set were simple LF words (e.g., PAGANA, ‘pagan’). The first set of pseudo-morphemic words (e.g., PELERA) were made from a real HF root (e.g., PEL-) and a real suffix (e.g., -ERA); the second set of pseudowords were made from a real LF root (e.g., TEJ-) and a real suffix (e.g., -UDO); and the simple pseudowords were created from real LF words by changing one or two letters (e.g., PEMURA). In addition, 16 fillers were included. All sets of stimuli were matched by initial letter and phoneme, length in letters and syllables, root length in letters (for morphologically complex stimuli), syllable frequency, *N*-size, and imageability (all *p* > 0.05). For *N*-size and imageability variables, the BuscaPalabras database (Davis and Perea, 2005) was used; the dictionary of Alameda and Cuetos (1995) was used for syllable frequency. The values for manipulated and controlled variables are shown in **Table 2** and the experimental stimuli are given in Appendix A.

**Table 2:** Psycholinguistic characteristics of stimuli.

	Morph	Lex	Base	Let	Syll	*N*-size	1st Syll	2nd Syll	3th Syll	Imag
	Cond	Freq	Freq	Leng	Leng		Freq	Freq	Freq	
**Words**	HF base	2.7	186^∗∗^	6	3	2.6	3,325	1,723	7,817	0.84
	LF base	4.00	3.84^∗∗^	6	3	2.6	2,998	2,312	12,520	1.04
	S	3.16	NA	6	3	1	5,389	2,334	6,013	0.21
**Pseudowords**	HF base	NA	131^∗∗^	6	3	2.3	3,793	2,628	10,324	NA
	LF base	NA	5.00^∗∗^	6	3	1	5,086	3,498	10,512	NA
	S	NA	NA	6	3	1	4,486	1,885	8,133	NA

*Morph Cond, morphological condition; Lex Freq, lexical frequency; Let Leng, letter length; Syll Leng, syllable length; *N*-size, neighborhood size; Syll Freq, syllable frequency; Imag, imaginability, HF, High frequency, LF, low frequency, S, simple. NA, not applicable. ^∗∗^Significant difference between HF base and LF base.*

### Procedure

All the participants performed two tasks: a word naming (reading task) and spelling-to-dictation (writing task). We first conducted the word naming task, and 15 days later the spelling-to-dictation was carried out. The procedure of this experiment was approved by the Ethics Committee of the Department of Psychology of the University of Oviedo. Parental written consent was collected for all participants.

#### Reading Task

Stimuli were written in 22-point Arial font. Firstly, there appeared a blank screen for 500 ms; then, a black asterisk was presented in the center of a gray field screen for 500 ms; the stimulus appeared 500 ms after the asterisk and remained on the screen for 4,000 ms. Experimental stimuli and fillers were presented in two blocks of 26 stimuli each and appeared randomly in each block. The two blocks were separated by a pause, and preceded by four practice trials in order for the child to become familiar with the task. Children were 30 cm from the screen, and at the beginning of the test, it was explained that they had to read. We gave them the following instructions: ‘Words and pseudowords will appear on the computer screen. You will have to read them aloud as quickly as possible without making any mistakes.’ Participants completed the task during individual sessions that lasted approximately 15 min. The responses were recorded in .WAV files using DMDX (Forster and Forster, 2003). The recordings were subsequently analyzed using Praat software (Boersma and Weenink, 2017) through which we obtained accuracy, RL, RD, and RCSD from the resulting spectrograms.

#### Spelling-to-Dictation Task

Stimuli presentation and digital recording of the writing responses were controlled by Ductus (Guinet and Kandel, 2010). The experiment was run on an HP Mini laptop. A WACOM Intuos 5 graphic tablet connected to the computer and an Intuos Inking Pen were used to register the participants’ responses. Auditory stimuli for presentation were recorded by a female speaker with a Plantronics microphone and edited with Audacity software.

In the spelling-to-dictation task, each trial started with the simultaneous presentation of an auditory signal and a 500-ms fixation point. The auditory stimulus was presented 500 ms after the offset of the fixation point. Participants had to write the stimulus in lower case as quickly and as accurately as possible on a lined sheet of paper placed over the digitizer. When they finished a response, participants were instructed to hold the pen over the next line of the response sheet, but to avoid any contact with the paper. Then the experimenter clicked the left button of the mouse to start a new stimulus. The stimuli were presented in a quasi-randomized order; four lists, including the total number of stimuli, were created after randomizing the stimuli. Twelve participants (six with dyslexia and six controls) performed the same list. The experimental sessions were conducted for each participant individually in a quiet room and lasted around 15 min. Accuracy, WL, WD, and WCSD were considered for the statistical analysis.

## Results

For analyses of latencies and durations, we only included data from correct responses; mistakes (self-corrections, substitutions, regressions, omissions, false responses) and outliers were excluded from analyses. Data were analyzed by using generalized linear mixed-effects modeling with the lme4 package in R (R Core Team, 2012). Mixed-effects models allowed us to estimate both fixed effects—i.e., replicable effects of theoretical interest (group, lexicality, morphological condition)—and random effects—i.e., unexplained effects because of random variation between items or participants (Baayen et al., 2008). We incrementally added the predictor variables (group, lexicality, morphological condition) and interactions to the model to see whether or not the model was improved. Model fit was assessed using chi-squared tests on the log-likelihood values to compare different models. The most complex adjustment but the smallest BIC (Schwarz, 1978) and significant chi-squared test for the log-likelihood were retained. *F*-values from the ANOVAs of type III with Satterthwaite approximation for degrees of freedom are reported for fixed effects. When interactions were significant, *t*-tests were performed and the *p*-values were adjusted via the Bonferroni method.

For the analysis of accuracy, mixed effects logistic regression (used to model binary outcome variables) was performed using the lme4 package in R (R Core Team, 2012).

**Table 3** shows data for the reading task (percentage of accuracy, means and standard deviations of reading durations) in each condition for both groups, whilst **Table 4** shows data for the writing task. Following the analyses, the significant results are reported below.

**Table 3:** Reading measures (accuracy, reading latencies, reading durations, and critical segment durations) by condition and group.

		Control group				Dyslexia group			
		Accuracy	RL	RD	CSD	Accuracy	RL	RD	CSD
		% (*SD*)	ms (*SD*)	ms (*SD*)	ms (*SD*)	% (*SD*)	ms (*SD*)	ms (*SD*)	ms (*SD*)
**Words**	HF base	97.92 (0.30)	753 (215)	598 (141)	244 (59)	81.25 (0.90)	1,263 (413)	811 (242)	341 (130)
	LF base	97.22 (0.40)	789 (247)	593 (143)	303 (51)	75.69 (1.41)	1,331 (459)	770 (235)	476 (122)
	Simple	94.44 (0.50)	856 (297)	604 (153)	285 (55)	70.83 (1.43)	1,315 (438)	794 (253)	434 (147)
**Pseudowords**	HF base	95.13 (0.45)	844 (269)	608 (171)	287 (69)	67.36 (1.30)	1,299 (441)	763 (226)	406 (88)
	LF base	95.83 (0.52)	861 (313)	635 (176)	304 (78)	68.05 (1.60)	1,351 (421)	831 (246)	444 (134)
	Simple	93.75 (0.50)	902 (284)	615 (150)	314 (80)	66.66 (1.51)	1,356 (429)	902 (311)	528 (156)

**Table 4:** Writing measures (accuracy, writing latencies, writing durations, and critical segment durations) by condition and group.

		Control group				Dyslexia group			
		Accuracy	WL	WD	CSD	Accuracy	WL	WD	CSD
		% (*SD*)	ms (*SD*)	ms (*SD*)	ms (*SD*)	% (*SD*)	ms (*SD*)	ms (*SD*)	ms (*SD*)
**Words**	HF base	98.60 (0.10)	1,036 (236)	2,418 (544)	1,150 (293)	92.36 (0.50)	1,280 (402)	2,967 (960)	1,324 (401)
	LF base	95.13 (0.30)	1,040 (233)	2,349 (538)	1,171 (314)	80.55 (1.20)	1,306 (306)	3,023 (1139)	1,367 (420)
	Simple	93.05 (0.22)	1,063 (252)	2,387 (517)	1,269 (445)	87.50 (0.75)	1,357 (358)	3,135 (1175)	1,568 (602)
**Pseudowords**	HF base	89.58 (0.63)	1,034 (234)	2,258 (505)	1,149 (350)	89.58 (0.60)	1,287 (324)	2,831 (903)	1,326 (394)
	LF base	88.88 (0.67)	1,044 (261)	2,450 (548)	1,178 (297)	79.86 (1.21)	1,316 (287)	3,109 (1164)	1,399 (420)
	Simple	90.27 (0.60)	1,064 (254)	2,397 (508)	1,293 (299)	86.80 (0.79)	1,372 (388)	3,004 (1093)	1,583 (594)

### Reading

Excluding practice trials and fillers, a total of 1,728 responses were collected (864 from each group), where 265 (15.33%) of responses were discarded because they were mistakes or non-responses. Children with dyslexia gave rise to 230 discarded responses of 864 collected (26.62%); children without dyslexia executed only 35 (4.05%) discarded responses.

#### Accuracy in Reading

The mixed effects logistic regression analysis showed group effect (*p* = 0.000; Estimate = 2.359, *SE* = 0.323; *OR* = 0.094, *CI* = 0.050–0.180) and lexicality effect (*p* = 0.006, Estimate = 0.503, *SE* = 0.185; *OR* = 0.604, *CI* = 0.420–0.870), as the number of mistakes was bigger for the DYS group than for the CON group, and for pseudowords than for words. The morphological condition effect was close to significance between HF base stimuli and simple ones (*p* = 0.099; Estimate = 0.370, *SE* = 0.224; *OR* = 0.690, CI = 0.444–1.072), as there was a higher probability of committing mistakes in simple than in HF stimuli. The participants’ intercepts vary with an *SD* of 0.846 (participants effect, *p* = 0.200), the items’ intercepts with an *SD* of 0.322 (items effect, *p* = 0.000).

#### Reading Latencies

Reading latency is considered as the time from when the stimulus appears on the screen to when the participant begins to read—i.e., the time a participant takes to initiate the response (see **Table 3**).

We found a statistically significant effect of group [*F*(1,45) = 50.26, *p* = 0.000], as the CON group initiated responses significantly faster than the DYS group (Estimate = 495, *SE* = 69); of lexicality effect [*F*(1,32) = 5.38, *p* = 0.026], as reading latencies were bigger for pseudowords than for words (Estimate = 50, *SE* = 21); and of morphological condition [*F*(2,32) = 3.82, *p* = 0.032]. Pairwise comparisons showed significant differences between HF base and simple words [*t*(32) = -2.76, *p* = 0.028], as RL were higher for simple stimuli than for HF base ones (Estimate = 73, *SE* = 26). The participants’ intercepts vary with an *SD* of 236.62 (participants effect, *p* = 0.000); items’ intercepts have an *SD* of 47.35 (items effect, *p* = 0.000); the *SD* of error not accounted for was 275.23.

#### Reading Duration

Reading duration is the time a participant needs to read a word, the articulation time (see **Table 3**). When RD was considered, we found a group effect [*F*(1,45) = 19.24, *p* = 0.000], as the CON group took less time to articulate a response than the DYS group (Estimate = 211, *SE* = 48); lexicality effect [*F*(1,30) = 5.85, *p* = 0.021], where pseudowords took more time than words (Estimate = 34, *SE* = 14); group by morphological condition [*F*(2,1359) = 4.11, *p* = 0.016]; and group by lexicality by morphological condition [*F*(2,1358) = 6.857, *p* = 0.001]. Pairwise comparison indicated a significant difference between HF base pseudowords and simple pseudowords for the DYS group [*t*(54) = -3.96, *p* = 0.014], as they took more time reading simple pseudowords than HF base ones (Estimate = 111, *SE* = 28). The participants’ intercepts vary with an *SD* of 165.50 (participants effect, *p* = 0.000); items’ intercepts have an *SD* of 36.61 (items effect, *p* = 0.000); the *SD* of error not accounted for was 128.07.

#### Reading Critical Segment Duration

**FIGURE 1:**
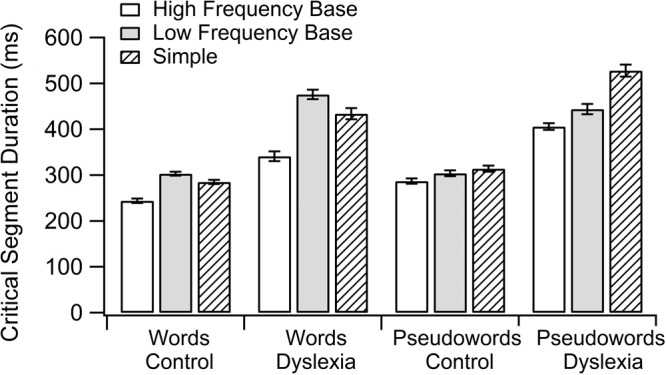
Group by lexicality by morphological condition interaction in reading critical segment duration.

Critical segment duration in reading was considered as the time needed to read a segment—in this case, the pronunciation of the first three phonemes of the stimuli, which correspond with the root in the morphological complex words (see **Table 3**). The results indicated a significant effect of group [*F*(1,45) = 25.42, *p* = 0.000], where children with dyslexia took more time than controls (Estimate = 111, *ES* = 22); of lexicality [*F*(1,30) = 6.40, *p* = 0.019], where pseudowords took more time than words (Estimate = 24, *ES* = 10); and of group by lexicality by morphological condition interaction [*F*(2,1343) = 4.19, *p* = 0.015]. In addition, group by morphological condition was close to significance [*F*(2,1343) = 2.52, *p* = 0.081]. As in the previous analysis, pairwise comparison indicated a significant difference between HF base pseudowords and simple pseudowords for the DYS group [*t*(49) = -3.69, *p* = 0.037], as they took more time to read simple pseudowords than HF base ones (Estimate = 72, *ES* = 19). The participants’ intercepts vary with an *SD* of 77.99 (participants effect, *p* = 0.000); items’ intercepts have an *SD* of 27.14 (items effect, *p* = 0.000); the *SD* of error not accounted for was 79.97 (see **Figure 1**).

### Spelling

Of 1,728 responses, we found 1,543 correct spellings and a total of 185 mistakes (10.70%), where the DYS group made 120 mistakes in 864 responses (13.89%) and the CON group made 65 (7.52%). Percentage and mean distribution of correct responses for participants in every morphological condition and group appear in **Table 4**.

#### Spelling Accuracy

After the mixed effects logistic regression analysis, we found a group effect (*p* = 0.011; Estimate = 0.722, *SE* = 0.286; *OR* = 0.485, *CI* = 0.276–0.851) and morphological condition, with significant differences between HF and LF stimuli (*p* = 0.012; Estimate = 0.782, *SE* = 0.31; *OR* = 0.457, *CI* = 0.31). The participants’ intercepts vary with an *SD* of 0.779 (participants effect, *p* = 0.000); the items’ intercepts have an *SD* of 0.558 (items effect, *p* = 0.000).

#### Written Latencies

Written latency is defined as the time between the onset of the stimulus and the occurrence of the first contact of the pen with the digitizing tablet (see **Table 4**).

Analyses showed a group effect [*F*(1,45) = 27.64, *p* = 0.000], as WL were higher for children with dyslexia than for children without dyslexia (Estimate = 269, *SE* = 51) and the morphological condition was close to significance [*F*(2,33) = 3.02, *p* = 0.06]. Pairwise comparisons showed that the differences tended to be significant between HF base and simple stimulus [*t*(33) = 2.39, *p* = 0.06; Estimate = 45, *SE* = 19]. The participants’ intercepts vary with an *SD* of 171.65 (participants effect, *p* = 0.000); the items’ intercepts have an *SD* of 25.57 (items effect, *p* = 0.09); the *SD* of error not accounted for was 252.29.

#### Writing Duration

Writing duration is the time between the first pen contact in producing a word and the last pen lift in the same word (see **Table 4**). After the analysis, we only found a group effect [*F*(1,45) = 12.40, *p = 0*.000], as children with dyslexia spent more time writing one stimulus than children without dyslexia (Estimate = 700, *SE* = 198). The participants’ intercepts vary with an *SD* of 682.00 (participants effect, *p* = 0.000); the items’ intercepts have an *SD* of 232.80 (items effect, *p* = 0.000); the *SD* of error not accounted for was 504.40.

#### Writing Critical Segment Duration

**FIGURE 2:**
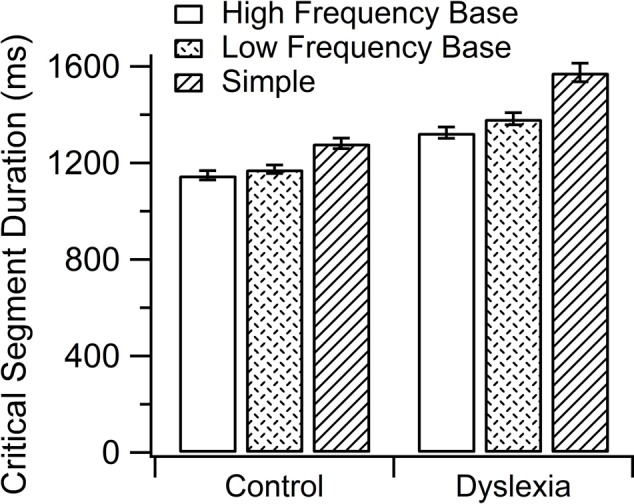
Group by morphological condition interaction in writing critical segment duration.

In this study, writing critical segment duration refers to the time between the first contact of the pen with the digitizer in a given stimulus and the beginning of the fourth letter of that stimulus (see **Table 4**). The trajectory and tangential velocity were considered to isolate the critical segment using geometric (cuspids and curvature maxima) and kinematic (velocity minima) criteria, as proposed by Kandel and Valdois (2006). We found an effect of group [*F*(1,45) = 9.07, *p* = 0.004; Estimate = 275.64, *SE* = 91.49], as the DYS group took more time than the CON group; of morphological condition [*F*(2,32) = 5.75, *p* = 0.007], with significant differences between HF base and simple stimuli [*t*(32) = -3.29, *p* = 0.007; Estimate = 199.72, *SE* = 60.55]; and LF base and simple stimuli were close to significance [*t*(33) = -2.34, *p* = 0.07; Estimate = 141.86, *SE* = 60.65]. We also found group by morphological condition interaction [*F*(2,1396) = 4.31, *p* = 0.013]. Pairwise comparisons showed significant differences between HF base and simple words in the DYS group [*t*(38) = -3.93, *p* = 0.005; Estimate = 248.00, *SE* = 63.01]. The participants’ intercepts vary with an *SD* of 313.00 (participants effect, *p* = 0.000); the items’ intercepts have an *SD* of 142.70 (items effect, *p* = 0.000); the *SD* of error not accounted for was 260.50 (see **Figure 2**).

## Discussion

In the present study, we addressed the role of morphology in both reading and spelling in Spanish children with dyslexia. We aimed to know if they access intermediate linguistic units (morphemes), improving their reading and spelling performance (accuracy and speed). In addition, we aimed to know whether facilitation could depend on the base (root) frequency or not. To achieve the objective, participants performed two tasks, word naming and spelling-to-dictation, where lexicality and morphological condition were manipulated. Different measures (accuracy and chronometric measures of the responses) were collected. In Spanish, the consistency of grapheme-to-phoneme mapping is sufficient to achieve the correct pronunciation when reading, and quite sufficient when spelling. Because the main interest was to test the effect of the base frequency of words, in this work we only used consistent phoneme–grapheme correspondences. In this sense, morphology probably would not be as necessary to achieve reading and spelling accuracy, but it would have an impact on spelling and reading speed.

In reading, results indicated that children with dyslexia took advantage of morphology, which is evident in the reading speed scores, as in Spanish reading accuracy is a measure less sensitive than reading speed. All the children initiated an earlier response (RL) when they faced HF base stimuli (words and pseudowords) than when they had to read a simple one. Both DYS and CON groups benefit from the HF base of stimulus to initiate the response of LF words and pseudowords. It is supposed that children, especially children with dyslexia, do not have robust orthographic representation of LF words and, of course, they have no representations of pseudowords, as they do not exist. But they showed longer RL for simple words than for HF base words, indicating that they were relying on shorter reading units (morphemes). We did not find benefits from the LF base of words in RL, so probably they do not have representations of small grain-size units with LF. Effects of morphology in RL in children with dyslexia were previously reported, but modulated by other variables, such as reading skills and lexical frequency (Burani et al., 2002, 2008; Marcolini et al., 2011; Suárez-Coalla and Cuetos, 2013). Regarding base frequency effects, it has been reported that HF base speeds up LF word reading in Italian adults (Colombo and Burani, 2002) and facilitates reading accuracy (pseudowords and LF words) in third grade children, both typically developing children and children with dyslexia (Angelelli et al., 2017). Otherwise, different results were described by Lázaro et al. (2013), where only eight year old skilled readers benefited from base frequency to respond in a lexical decision task. This makes sense, though, considering the nature of the tasks. In a lexical decision task, it is necessary to process both constituents (root and suffix) to make a decision, involving a time cost. In contrast, in a word naming task, RL is the time a participant takes to initiate the response, so children could rely on the initial lexical unit (root) to start pronunciation, without processing the whole stimulus. In this sense, when they are able to recognize the HF base root they start to pronounce the words, even if they do not have the orthographic representation of the whole word.

It should be noted that the most important results concerned RD and RCSD, which were not considered in previous studies about morphology. RD was modulated by readers and lexical-morphological condition, as differences were only found between HF base and simple pseudowords in the DYS group. The same pattern was found when RCSD was considered, as the DYS group spent less time on the articulation of the segment when it was an HF base one in a pseudo-morphemic word. These results suggest that children with dyslexia are able to use morphemic pre-assembled representations, with effects on articulation durations. According to this, morphological information is a useful mechanism to gain reading speed in this population. It probably cannot be said that the advantage is due to the semantic access to the morphological units, taking into account the task performed. However, according to grain-size theory, children could be relying on salient units of different size (Ziegler and Goswami, 2005). It is noteworthy that we did not find the morphological effect in RD in the CON group, nor in word reading in the DYS group, which could signify that children process the whole stimulus before they start to read.

As for spelling, children with dyslexia committed more mistakes and were slower than children without dyslexia. However, both the DYS and CON groups benefited from morphology, as accuracy was higher for HF base stimuli than for LF base ones and they tended to start to spell HF base stimuli faster than simple ones. That implies that morphological information (base frequency) supposes an advantage for them when they have to spell. This is consistent with recent studies, supporting the idea that morphological structure favors spelling accuracy in opaque (Bourassa et al., 2006; Quémart and Casalis, 2016) and transparent orthographies (Angelelli et al., 2017). In the present study, there were no phoneme-to-grapheme inconsistencies, but an advantage of morphology was found. More interestingly, when WCSD was considered, morphological effects were modulated by group, where the DYS group spent less time spelling an HF base critical segment than a simple one. These data seem to indicate that children with dyslexia have representation of chunks of letters (morphemes) that help them to improve speed of handwriting unfamiliar stimuli. Taking together the results of WL and WCSD, we can observe that the morphological effects in WL continue in WCSD, but only for spelling in children with dyslexia. This might suggest that the CON group do not rely on morphology after recovering the orthographic output. In addition, the influence of morphology on the DYS group handwriting reinforces the hypothesis, suggested by Sumner et al. (2014), that their slowness is due to linguistic factors rather than the existence of graphomotor speed problems. Evidence reported here can be easily integrated into the context of the role of morphology in handwriting, a process mediated by linguistic units and spelling ability, independent of the semantic level (Orliaguet and Boë, 1993; Kandel et al., 2008, 2012).

Recently, Quémart and Lambert (2017) explored the influence of morphological structure on French adults’ and children’s handwriting. They also compared morphologically complex and simple words, and adults exhibited shorter latencies for morphologically complex words than for simples ones. However, children did not show effects of morphological structure in writing latencies, suggesting that morphological effects are modulated by expertise in the written language. In contrast, our data support the effect of morphology in both typically developing children and children with dyslexia, but the differences only tended to be significant between HF base and simple stimulus. The discrepant results could be due to the task, as Quémart and Lambert (2017) used a copy instead of a spelling-to-dictation task. The processes prior to motor execution vary from one task to another, and possibly spelling-to-dictation could benefit more from the morphological structure of words than copying. Another explanation could be related to the orthographic system, as French is more opaque than Spanish, and the orthographic system could determine the influence of the morphological structure according to writing exposure. Additional studies are needed to look more deeply into these aspects.

Furthermore, it is interesting to highlight another important result considering the outcomes of the two tasks (reading and writing). The HF base speeds up RL and WL in both groups, but morphological condition continues to influence RCSD and WCSD only in the group with dyslexia. This result suggests that morphemes could be programmed before writing or reading begins in typically developing children, but the influence continues after the start of reading or writing in children with dyslexia. Additionally, the absence of a lexicality effect in writing suggests that children (in this study) process LF words and pseudowords in a similar way. However, further research is necessary to know differences between reading and writing of morphologically complex stimuli.

In summary, the evidence achieved in the present study suggests that children with dyslexia benefit from morphological structure, especially an HF base, in reading and spelling. Children with dyslexia speed up in their reading and spelling of unfamiliar stimuli when they involve an HF base. That suggests that they are familiarized with letter chunks that constitute an HF morpheme. The results support important implications for teaching and clinical work with children with dyslexia. Taking into consideration difficulties they exhibit in developing orthographic representations of the whole word, it could be interesting to encourage the use of units larger than graphemes and help them to develop morphological processing.

Finally, a limitation of this study was the small number of stimuli for each experimental condition. The reason for such a selection was that children with dyslexia show fatigue after long reading and writing tasks. Consequently, when many stimuli are used, the results may not be reliable. However, it would be useful to confirm the results of this study with new stimuli and new participants.

## Author Contributions

PS-C and FC developed the study concept and design. CM-G performed testing and data collection. PS-C and FC analyzed data and drafted the manuscript.

## Conflict of Interest Statement

The authors declare that the research was conducted in the absence of any commercial or financial relationships that could be construed as a potential conflict of interest.
