# Supplementary material for: Morpheme-Based Reading and Writing in Spanish Children with Dyslexia
Source: Front Psychol. 2017 Nov 7;8:1952. doi: 10.3389/fpsyg.2017.01952 (PMC5682102; doi:10.3389/fpsyg.2017.01952)
Supplement: Supplementary file 1 [file Data_Sheet_1.pdf]

Appendix A.

Words			Pseudowords		
HF base	LF base	Simple	HF base	LF base	Simple
manojo	morera	maraca	manero	moreta	mafoca
meseta	mulero	mofeta	mesoso	mulado	moseta
peludo	poroso	pagana	pelera	porazo	pemura
penoso	puñado	pagoda	peneda	puñojo	pocala
tipazo	tarada	tenaza	tipeda	taroso	pogana
tonada	tejado	tesoro	tonado	tejudo	tegoro
